# Half-lives of PAHs and temporal microbiota changes in commonly used urban landscaping materials

**DOI:** 10.7717/peerj.4508

**Published:** 2018-03-19

**Authors:** Marja I. Roslund, Mira Grönroos, Anna-Lea Rantalainen, Ari Jumpponen, Martin Romantschuk, Anirudra Parajuli, Heikki Hyöty, Olli Laitinen, Aki Sinkkonen

**Affiliations:** 1 Faculty of Biological and Environmental Sciences, Ecosystems and Environment Research Programme, University of Helsinki, Lahti, Finland; 2 Division of Biology, Kansas State University, Kansas, Manhattan, United States of America; 3 Faculty of Medicine and Life Sciences, University of Tampere, Tampere, Finland

**Keywords:** Polycyclic aromatic hydrocarbons, Urban pollution, Biodegradation, Microbial community composition, Human health, Health-associated bacteria

## Abstract

**Background:**

Polycyclic aromatic hydrocarbons (PAHs) accumulate in urban soils, and PAH contamination can change soil microbial community composition. Environmental microbiota is associated with human commensal microbiota, immune system and health. Therefore, studies investigating the degradation of PAHs, and the consequences of soil pollution on microbial communities in urban landscaping materials, are crucial.

**Methods:**

Four landscaping materials (organic matter 1, 2, 13 and 56%) were contaminated with PAHs commonly found at urban sites (phenanthrene, fluoranthene, pyrene, chrysene and benzo(b)fluoranthene) in PAH concentrations that reflect urban soils in Finland (2.4 µg g^ -1^ soil dry weight). PAHs were analyzed initially and after 2, 4, 8 and 12 weeks by gas chromatography-mass spectrometry. Half-lives of PAHs were determined based on 12-weeks degradation. Bacterial communities were analyzed at 1 and 12 weeks after contamination using Illumina MiSeq 16S rRNA gene metabarcoding.

**Results:**

Half-lives ranged from 1.5 to 4.4 weeks for PAHs with relatively low molecular weights (phenanthrene, fluoranthene and pyrene) in landscaping materials containing 1–2% organic matter. In contrast, in materials containing 13% and 56% organic matter, the half-lives ranged from 2.5 to 52 weeks. Shorter half-lives of phenanthrene and fluoranthene were thus associated with low organic matter content. The half-life of pyrene was inversely related to the relative abundance of Beta-, Delta- and Gammaproteobacteria, and diversity of Bacteroidetes and Betaprotebacteria. Compounds with higher molecular weights followed compound-specific patterns. Benzo(b)fluoranthene was resistant to degradation and half-life of chrysene was shorter when the relative abundance of Betaproteobacteria was high. Temporal microbiota changes involved increase in the relative abundance of Deltaproteobacteria and decrease in genera *Flavobacterium* and *Rhodanobacter*. Exposure to PAHs seems to adjust microbial community composition, particularly within class Beta- and Deltaproteobacteria.

**Conclusions:**

In this study, PAH degradation depended on the organic matter content and bacterial community composition of landscaping materials. Contamination seems to alter bacterial community composition in landscaping materials depending on material type. This alteration includes changes in bacterial phyla associated with human health and immune system. This may open new possibilities for managing urban environments by careful selection of landscaping materials, to benefit health and wellbeing.

## Introduction

Polycyclic aromatic hydrocarbons (PAHs) have been classified as priority environmental pollutants by the United States Environmental Protection Agency (USEPA) and by the European Environment Agency (EEA). PAHs constitute a large class of fused aromatic ring compounds that are present in petroleum products and formed from the incomplete combustion of fossil fuels and biomass (Boström et al., 2002). Combustion of fossil fuels is the most important source of PAHs in urban environments, and PAH exposure in large cities can be very high (Boström et al., 2002; Ravindra, Sokhi & Van Grieken, 2008; Hong et al., 2016). Airborne PAHs often accumulate in soils (Haritash & Kaushik, 2009) and the concentrations in urban soils can be 10–100 times higher than in unpolluted rural soils (Hiller et al., 2015). Microbial degradation of PAHs can be significant in soil depending on soil microbial activity and other factors, such as soil properties, climate and bioavailability of PAHs (Koivula et al., 2004; Haritash & Kaushik, 2009; Crampon et al., 2014). Besides microbial activity, other pathways for PAHs in the environment include volatilization, photo-oxidation, chemical oxidation, sorption to soil particles and leaching (Haritash & Kaushik, 2009).

Many bacterial species degrade PAHs, but only a limited number of bacteria degrade PAHs with five or more aromatic rings, mainly because of their low bioavailability (Johnsen, Wick & Harms, 2005). However, PAHs with high molecular weight may be degraded in a series of processes by consortia of microbes, such as bacteria and fungi (Johnsen, Wick & Harms, 2005). Many members of the phylum Proteobacteria degrade PAHs, and they are more abundant in PAH polluted soils compared to non-contaminated soils (Singleton et al., 2006; Lors et al., 2010; Martin et al., 2012; Parajuli et al., 2017). Typically, PAH contamination changes the abundances within the soil microbial communities, specifically including phyla Proteobacteria, Actinobacteria and Bacteroidetes (Lors et al., 2010; Martin et al., 2012; Peng et al., 2013; Mukherjee et al., 2014; Sawulski, Clipson & Doyle, 2014; Ren et al., 2015; Parajuli et al., 2017). For example, Mukherjee et al. (2014) reported that proteobacteria, particularly Betaproteobacteria and Gammaproteobacteria, dominated in the zones with high PAH pollution and the abundance of Actinobacteria declined in these sites, whereas Sawulski, Clipson & Doyle (2014) reported that Actinobacteria dominate in fluoranthene polluted soil, but compared to unpolluted soil more Gammaproteobacteria and less Bacteroidetes were identified in PAH polluted soil.

PAH exposure causes a variety of health risks, ranging from carcinogenic and mutagenic changes to respiratory problems, hematological changes and neurological dysfunctions, and PAHs may induce immune-suppression in humans (Kim et al., 2013; Kamal et al., 2015). In addition to the direct health consequences, PAHs may also change the abundance and diversity of human health-associated bacteria (Parajuli et al., 2017). Since the highest soil PAH concentrations are usually found in urban centers where source intensity is highest (Heywood et al., 2006; Haugland, Ottesen & Volden, 2008; Hiller et al., 2015), microbial communities in urban soils are likely affected by PAH pollution. Altered environmental microbial communities may lead to changes in human commensal microbiota, in turn affecting the human immune system (Ege et al., 2012; Hanski et al., 2012; Heederik & Mutius, 2012; Ruokolainen et al., 2015). Many factors affect the composition of human commensal microbiota, and skin microbiota may differ between rural and urban children and this microbial composition is associated with allergic sensitization (Hanski et al., 2012; Lehtimäki et al., 2017). Despite this, studies connecting PAH pollution, microbial community composition in urban soils and health-associated bacteria are lacking.

Urban areas are often characterized by a high degree of artificial surfaces, both paved surfaces (asphalt, buildings etc.) and non-sealed surfaces (man-made gardening and landscaping materials) (Artmann, 2014; Morabito et al., 2016). Urban soil often differs drastically in composition and function from natural soils (Morel, Chenu & Lorenz, 2015). For example, due to mechanical compaction, urban soils have a high bulk density and can be composed of coarse anthropogenic materials with reduced biological activity (Morel, Chenu & Lorenz, 2015). Since more than half of the global human population lives in urban areas (United Nations, 2014), it is surprising how little attention urban landscaping materials have gained in pollution-oriented studies. Furthermore, laboratory studies with PAH contamination have often been carried out with unrealistically high concentrations rarely found in urban soils (Johnsen, Wick & Harms, 2005; Crampon et al., 2014). Therefore, there is a need for studies to investigate how fast realistic urban concentrations of pollutants are degraded in different landscaping materials, and what changes pollutants cause in bacterial communities in those materials.

In this study, we analyzed the bacterial community composition and diversity changes in landscaping materials contaminated with five different PAHs in a 12-week laboratory experiment. We specifically focused on PAH concentrations similar to those common in polluted urban soil. We hypothesized that the half-life and degradation of PAHs is connected to soil properties such as organic matter content and the pH of the landscaping materials. In addition, we sought to find potential associations between bacterial community composition and PAH degradation, and to estimate how much PAH degradation depends on the number of aromatic rings. We also hypothesized that realistic urban concentrations of PAHs cause changes in the microbial community composition in commonly used landscaping materials, and that these changes differ between different landscaping materials. We particularly expected to observe changes in the bacterial community composition, diversity and relative abundance of Actinobacteria, Bacteroidetes and Proteobacteria. Finally, we discuss bacterial community composition changes of human health-associated bacteria.

The type of landscaping material is of great importance, both for the PAH degradation in an urban environment and for the exposure of urban inhabitants to environmental microbes important for the human immune system. The current study provides information about landscaping materials for urban planners to build sustainable cities suitable for human health and wellbeing.

## Materials and Methods

### Selection of landscaping materials

Four different landscaping materials were included in the study: Coarse peat-sand (Kekkilä Group, Vantaa, Finland), fine peat-sand (Kekkilä Group, Vantaa, Finland), gardening compost (Biolan, Eura, Finland) and sandy gravel of glacial origin (Rudus, Lahti, Finland) (Table S1). The selected landscaping materials represent the whole spectrum of the most widely used materials in Finland, ranging from microbiologically rich compost material to mineral aggregate fraction with a low microbial activity.

### Physicochemical analyses of materials

The organic matter content of each material was determined by a standardized loss-on-ignition method (SFS 3008, 1990). Moisture content was measured by drying samples in an oven (+105 °C) to constant weight and the water-holding capacity was determined with the method of Priha & Smolander (1999) and pH according to the SFS-ISO 10390 (2005) standard. Bioavailable nutrients (nitrate NO${}_{3}^{-}$, ammonium NH${}_{4}^{+}$ and phosphate PO${}_{4}^{3-}$) were extracted and filtered (Whatman^®^ 5892, White ribbon, by Schleichner and Schuell) as described by Sinkkonen et al. (2013) using the QuikChem 8000 flow injection analysis system (LACHAT Instruments Inc., Loveland, CO, USA). Approximately 10 g of fresh landscaping material was dried and crushed to measure elements (Al, Cu, Fe, Mn, P, Pb) with Inductive Coupled Plasma Mass Spectrometry (ICP-MS, Elan 6000; Perkin Elmer Inc., Waltham, MA, USA), and to measure carbon and nitrogen with the LECO C/N/S-200-analyzator (Leco Corporation, Saint Joseph, MI, USA).

### PAH degradation experiment

There were five replicates (500 ml glass pots with lids and passive aeration, 100 g dw per pot) per landscaping material that were contaminated artificially with a total of 2.4 µg g^−1^ dw of PAHs phenanthrene, fluoranthene, pyrene, chrysene and benzo[b]fluoranthene (Table S2). These five PAHs are often found from urban sites (Boström et al., 2002; Saarnio et al., 2008; Tarvainen et al., 2013). Contamination level for phenanthrene, fluoranthene and pyrene was 0.6 µg g^−1^, and for chrysene and benzo[b]fluoranthene it was 0.3 µg g^−1^. Total PAH concentrations were similar to those in man-made urban soils in Finland (approximately 0.3–3.6 µg g^−1^, mean 1.3 ± 1.2) (Tarvainen et al., 2013). For PAH measurements, one non-contaminated control replicate was included for each landscaping material to measure background PAH levels, i.e., to ensure that materials were uncontaminated.

Before mixing PAHs, each landscaping material was homogenized, sieved (ø 0.5 mm), and rehydrated to 60% of its water-holding capacity (Table S1). An aliquot (2 g) of each material (five replicates) was spiked with a total concentration of Σ5PAHs = 120 µg g^−1^ diluted in acetone. Non-contaminated replicates were spiked with acetone. For bacterial sequencing, five 2 g subsamples were randomly drawn (five sampling points from each non-contaminated control at the depth of 1–6 cm). The five subsamples were combined to generate a separate non-contaminated sample per each landscaping material that represented bacterial community composition in non-contaminated conditions. This method has been shown to be able to detect the dominant members of the bacterial community in heterogeneous soil (Manter, Weir & Vivanco, 2010). The spiked materials were mixed and kept in a fume hood for 1 day to allow acetone to evaporate. Then, 2 × 2 g of each spiked material were added to 2 × 98 g (in dry equivalent) of the corresponding wet material and mixed carefully and divided in two to obtain the initial concentration (Table S3). Spiked materials were stored at 16 ± 1 °C in glass flasks (500 ml) with an air hole (ø 3 mm) in the dark and the moisture content was kept at 60% of their water-holding capacity. The experimental temperature represents an average temperature in the southern part of Finland during summer (Finnish Meteorological Institute, 2017). PAH analyses were carried out immediately after spiking and after four, eight and 12 weeks. Initial concentrations for chrysene and benzo[b]fluoranthene were confirmed after two weeks. This was done because the extractability, especially for high molecular weight PAHs, may decrease after two weeks (Luo et al., 2012). DNA was analyzed for bacterial (16S) communities at weeks 1 and 12 using Illumina MiSeq metabarcoding (Table S4).

### PAH analyses

PAH concentrations were determined using hexane and acetone mixture (1:1 v/v) extraction and analyzed as described by Honkonen & Rantalainen (2013) using gas chromatography-mass spectrometry (Shimadzu GC–MS-QP5000) system equipped with an AOC-20i autoinjector and a 30-m ZB-5MS column (0.25 mm i.d., 0.25 µm film thickness). The mass spectrometer interface temperature was set to 280 °C. The oven temperature program was set as follows: 80 °C for 1 min, 10 °C/min to 250 °C, 7 °C/min to 280 °C and 20 °C/min to final 320 °C for 5 min. We used a deuterated PAH mixture (naphthalene-*d*8, acenaphthene-*d*10, phenanthrene-*d*10, chrysene-*d*12 and perylene-*d*12) (Dr. Ehrenstorfer Gmbh, Germany) as a standard and Anthracene-*d*10 (Dr. Ehrenstorfer GmbH, Germany) as a recovery standard.

#### Kinetic modeling and estimation of half-life times

Kinetic analysis was used to determine the degradation of PAHs (Oleszczuk & Baran, 2003; Zahed et al., 2011). Half-life of PAHs was determined on the basis of the kinetic equation of the first order (Zahed et al., 2011).

(1) \begin{eqnarray*}C={C}_{0}{e}^{-kt}\end{eqnarray*}

where *C* is the PAH concentration (ng g^−1^) at time *t* (weeks) *C*_0_ isthe initial concentration of PAH (ng g^−1^) and *k* is rate constant of the change in the PAH concentration (week^−1^). Half-life times (*t*1∕2) were calculated by Eq. (2) (Oleszczuk & Baran, 2003; Zahed et al., 2011).

(2) \begin{eqnarray*}t1/2= \frac{\ln \nolimits 2}{k} \end{eqnarray*}

where *k* is the rate constant of Eq. (1). We also calculate the percent concentrations (*C*%) at each time point based on the decrease in the total concentration of PAHs using the following Eq. (3).

(3) \begin{eqnarray*}C\text{%}=100- \frac{(Ci-Cr)}{Ci} \ast 100\end{eqnarray*}

where *Ci* is the initial and *Cr* the residual PAH concentration.

### DNA extraction, PCR and Illumina MiSeq sequencing

Samples for MiSeq sequencing were prepared as in Veach, Dodds & Jumpponen (2015). Samples were stored in the deep freezer (<−70 °C) before DNA extraction. Total DNA was extracted from 0.25–0.3 g of materials using a PowerSoil^®^ DNA Isolation Kit (MoBio Laboratories, Inc., Carlsbad, CA, USA) according to the manufacturer standard protocol. Sterile water was used as negative control. DNA was checked with agarose gel (1.5%) electrophoresis. Total DNA concentration was measured with Quant-iT™ PicoGreen^®^ dsDNA reagent kit (Thermo Fisher Scientific, Waltham, MA, USA). The DNA concentration was adjusted to 0.35–0.4 ng/µl for each sample. DNA was analyzed for bacterial (16S) communities using a two-step PCR approach to avoid a 3′-end amplification bias resulting from the sample-specific DNA tags (Berry et al., 2011). The V4 region within the 16S ribosomal RNA (rRNA) gene was amplified by primary PCR (three replicates from each sample) using 505F and 806R primers (Caporaso et al., 2012). Primary PCR was carried out in a reaction mixture (reaction volume 50 µl) consisting of 1 µl each of 10 mM deoxynucleoside triphosphates (dNTPs; Thermo scientific, MA, USA) 5 µl forward primer 505F (10 µM; 5′-GTGCCAGCMGCCGCGGTAA-3′) and 5 µl reverse primer 806R (10 µM; 5′-GGACTACHVGGGTWTCTAAT-3′), 0.5 µl 2 U/µl Phusion Green Hot Start II High-Fidelity DNA polymerase (Thermo Fisher Scientific, Waltham, MA, USA), 10 µl 5× Green HF PCR buffer (F-537), 5 µl template DNA and 23.5 µl sterile water. The PCR reaction was run in a thermocycler (MJ Research, Waltham, MA, USA) as follows: initial denaturation at 98 °C for 5 min, followed by 25 cycles with denaturation at 94 °C for 1 min, annealing for 10 sec at 50 °C and extension for 1 min at 72 °C, and then a final extension at 72°C for 10 min. A positive control (*Cupriavidus necator* JMP134, DSM 4058) was included in PRC to ensure that the PCR worked, and a negative control (sterile water) to detect possible contaminations. DNA was detected with agarose gel (1.5%) electrophoresis. The PCR products were purified using Agencourt AMPure XP solution (Beckman Coulter Inc., Brea, CA, USA) (1:1 ratio of bead solution to PCR volume) to minimize the carryover of primary PCR primers. Three replicates of the cleaned amplicons were pooled and diluted 1:5.

Cleaned and diluted primary PCR products were targeted in the secondary PCR (TagPCR). Reaction mixture to the TagPCR was equal as above except reverse primer included a 12 bp unique Multiplexing Identifier tag (MID-806R). The amplification program was the same as above except that there were only ten cycles with initial denaturation and final extension. TagPCR products were visualized on an agarose gel, purified with Agencourt AMPure and three replicates were pooled similarly to primary PCRs. DNA concentration for each sample was measured with PicoGreen and samples were pooled to equal amounts–150 ng. The sequencing was performed at the Kansas State University using Illumina MiSeq platform with a 2 ×300 bp version 3 kit sequencing kit according to manufacturer’s protocol. The GeneRead DNA Library I Core Kit (catalog # 180432; Qiagen, Hilden, Germany) was used to ligate Illumina’s TruSeq adapters to amplicons.

### Bioinformatics

Raw sequencing data was processed using Mothur (version 1.38.1, Schloss et al., 2009). The sequence processing protocol partly followed the pipeline suggested by Schloss, Gevers & Westcott (2011) and Kozich et al. (2013). The paired sequences contained in reverse and forward fastq files were aligned into a contig. Sequences were trimmed and screened to remove any that had mismatches with primer or DNA-tag sequences, ambiguous bases or homopolymers larger than 8 bp long.

Bacterial sequences were aligned against a SILVA reference (version 123, Pruesse et al., 2007), preclustered to minimize sequencing errors (Huse et al., 2010) and screened for chimeras with UCHIME (Edgar et al., 2011), which uses the abundant sequences as a reference. The chimeric sequences were removed and non-chimeric sequences were classified using the Mothur version of Bayesian classifier (Wang et al., 2007) with the RDP training set version 14 (Cole et al., 2009) with 80% bootstrap threshold. Sequences were removed from the analyses if classified to Chloroplast, Mitochondria, unknown, Archaea or Eukaryota.

A pairwise distance matrix for unique sequences was calculated and OTUs clustered at 97% sequence similarity using nearest neighbor (single linkage) joining. All bacterial sequence data was accessioned into the Sequence Read Archive (Sequence read Archive Accession SRX3195289–SRX3195338). Low abundance OTUs (≤2) were removed. Sequence profiles revealed similar taxonomic classifications between negative controls and sandy gravel, which is typical with samples containing a low microbial biomass (Salter et al., 2014). Altogether 14 abundant OTUs detected in negative controls were removed from sequence data and sandy gravel was discarded from community composition analyses because of the questionable sequence reads for bacteria. Other samples were subsampled to the smallest sample sequence depth at 6374 sequences for community composition analyses. Good’s coverage index (average ± SD: 0.93 ± 0.04) was used to determine OTU coverage adequacy for diversity and community composition analyzes. OTU richness (the Chao index) and diversity (the Shannon index) were calculated with summary.single command (Table S5).

### Statistics

**Chemical dissipation.** Multivariate analysis of variance (MANOVA) with Bonferroni correction in SPSS (v24) was used to compare half-life of PAHs (log transformed) in different landscaping materials. The linear regression models were used to estimate the relationship between half-lives and properties of the landscaping materials, and between half-lives and bacteria relative abundances and diversities.

**Bacterial community analysis.** PERMANOVA (R v3.4.0, R Core Team, 2017, function *adonis* in *vegan* with Bray-Curtis metric) was used to compare temporal differences in the bacterial community composition between weeks 1 and 12. The non-metric multidimensional scaling (NMDS) in R was used to visualize the difference in bacterial community composition (*meta* MDS function in the vegan package). Bray-Curtis distance matrices (R-package *vegan*) were used to compare each non-contaminated pot with the five contaminated pots within each landscaping material. In detail, Bray-Curtis distances were calculated between composite non-contaminated sample and contaminated samples. This resulted in five Bray-Curtis distances per landscaping material within each time point. Shannon diversity index for bacterial phylotypes was determined by using the function *diversity* in the *vegan* package. Temporal microbiota changes were determined for dominant phyla (Actinobacteria, Bacteroidetes and Proteobacteria) and classes (Alpha-, Beta-, Delta and Gammaproteobacteria), and to genera which abundance was more than 1%. Finally, the differences in the richness (the Chao index), diversity (the Shannon index) and relative abundances between weeks 1 and 12 were determined using the paired *t*-test in R.

## Results

### Degradation of PAHs

Recovery of phenanthrene from freshly spiked sandy gravel ranged from 30% to 42%, indicating that phenanthrene evaporated from sandy gravel when acetone was permitted to evaporate in the fume hood. Other recoveries from freshly spiked materials were on average 101 ± 15% for phenanthrene, 105 ± 12% for fluoranthene, 110 ± 13% for pyrene, 91 ± 17% for chrysene and 113 ± 21% for benzo[b]fluoranthene (Table S3). Recoveries for benzo[b]fluoranthene were very high at week 2 (171 ± 37) compared to the initial recoveries and they were still high after 12 weeks (114 ± 15). Consequently, half-life for benzo[b]fluoranthene cannot be reasonably calculated. Surrogate standard recoveries were, on average, 77 ± 17% for all PAH analyzes (Table S3).

For the three PAHs with molecular weight ≤202 g mol^−1^ (phenanthrene, fluoranthene and pyrene), the half-lives were shorter in coarse mineral soils with organic content of 1–2% (sandy gravel and coarse peat-sand) than in soils with organic content of 13% (fine peat-sand) and 56% (gardening compost) (MANOVA: *F* ≥ 92, *df* = 3, *p* < 0.001; Table 1, Table S6). In addition, half-life of pyrene was 36 weeks shorter in gardening compost than in fine peat-sand (*F* = 101, *df* = 3, *p* < 0.001; Table 1, Table S6). Organic matter content was directly related to the half-life of phenanthrene (*F* = 13, *R* = 0.65, *r*^2^ = 0.43, *df* = 18, *p* < 0.01) and fluoranthene (*F* = 21, *R* = 0.74, *r*^2^ = 0.55, *df* = 17, *p* < 0.001) (Table S7). Half-life of pyrene was inversely related to the relative abundance of Beta-, Delta- and Gammaproteobacteria, and directly to the abundance of Actinobacteria and Alphaproteobacteria (Table 2). In addition, diversity of Bacteroidetes and Betaproteobacteria was inversely related to the half-life of pyrene (Table 2).

**Figure 1:**
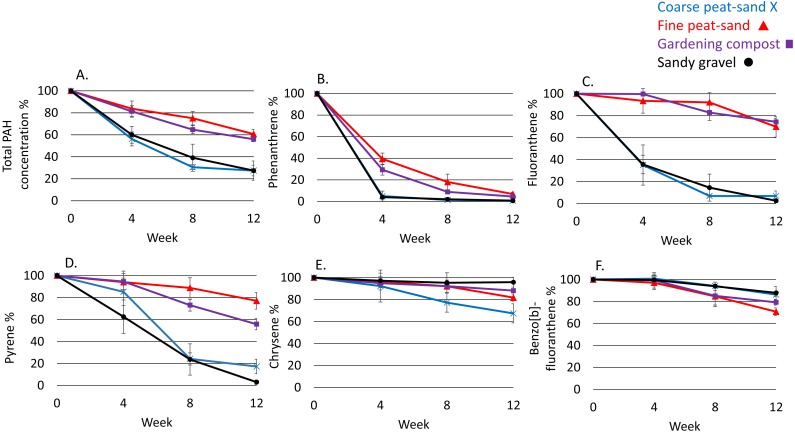
Decrease of PAHs. %-decrease of PAHs in the studied landscaping materials (mean ± SD): (A) Total PAH concentration. (B) Phenanthrene. (C) Fluoranthene. (D) Pyrene. (E) Chrysene. (F) benzo[b]fluoranthene.

The two high molecular weight PAHs (≥228 g mol^−1^: Chrysene and benzo[b] fluoranthene) were more persistent than the three compounds with lower molecular weights (Fig. 1). Half-life of chrysene was more than 3 times longer in sandy gravel than in other landscaping materials (MANOVA: *F* = 18, *df* = 3, *p* < 0.01; Table S6). Half-life of chrysene was inversely related to the relative abundance of Proteobacteria (*F* = 6, *df* = 11, *R* = 0.59, *r*^2^ = 0.35, *p* < 0.05), particularly Betaproteobacteria (*F* = 8, *df* = 11, *R* = 0.64, *r*^2^ = 0.41, *p* < 0.02) (Table S7).

**Table 1:** Half-lives of PAHs were determined based on 12 week’s degradation. Half-lives are shown in weeks (Mean ± SD, *n*, number of replicates).

	Coarse peat-sand	Fine peat-sand	Gardening compost	Sandy gravel
Phenanthrene	1.5 ± 0.1 (*n* = 5)	2.8 ± 0.2 (*n* = 5)	2.5 ± 0.1 (*n* = 5)	1.5 ± 0.1 (*n* = 5)
Fluoranthene	2.4 ± 0.2 (*n* = 4)	39 ± 3.1 (*n* = 4)	36 ± 7.0 (*n* = 5)	2.8 ± 1.0 (*n* = 5)
Pyrene	4.4 ± 0.6 (*n* = 4)	52 ± 26 (*n* = 5)	16 ± 1.8 (*n* = 5)	2.7 ± 0.6 (*n* = 5)
Chrysene	22 ± 4.7 (*n* = 4)	49 ± 6.5 (*n* = 5)	56 ± 18 (*n* = 4)	198 ± 102 (*n* = 4)
Sum of PAHs	5.6 ± 0.3 (*n* = 4)	17 ± 0.7 (*n* = 4)	14 ± 1.1 (*n* = 5)	6.4 ± 1.6 (*n* = 5)

**Table 2:** Regression between half-life of pyrene and relative abundances and diversities of bacterial phyla and class. Half-life of pyrene correlated inversely with relative abundance of Beta-, Delta- and Gammaproteobacteria, and directly with the relative abundance of Actinobacteria and Alphaproteobacteria. In addition, regression was found between lower half-life of pyrene and higher diversity of Bacteroidetes and Betaproteobacteria.

	*df*	*F*	*R*	*r*^2^	*t*	*p*-value
**Abundance**						
Actinobacteria	13	9	0.64	0.41	3.0	0.010
Alphaproteobacteria	13	12	0.69	0.48	3.5	0.004
Betaproteobacteria	13	7	0.60	0.36	−2.7	0.018
Deltaproteobacteria	13	10	0.67	0.44	−3.2	0.007
Gammaproteobacteria	13	6	0.57	0.32	−2.5	0.027
**Diversity**						
Bacteroidetes	13	18	0.76	0.58	−4.3	0.001
Betaproteobacteria	13	7	0.60	0.36	−2.7	0.018

**Notes.**

*r*^2^ Coefficient of Determination *df* Degree of Freedom *R* Correlation Coefficient *t* *t*-test statistic

### Bacterial community compositions

Three of the four landscaping materials contained detectable levels of bacterial sequence. Based on Illumina sequencing data, the rarefied data had 11,557 OTUs and an average of 27,463 bacteria sequences for coarse peat-sand, 79,943 for fine peat-sand and 58,600 for gardening compost.

**Figure 2:**
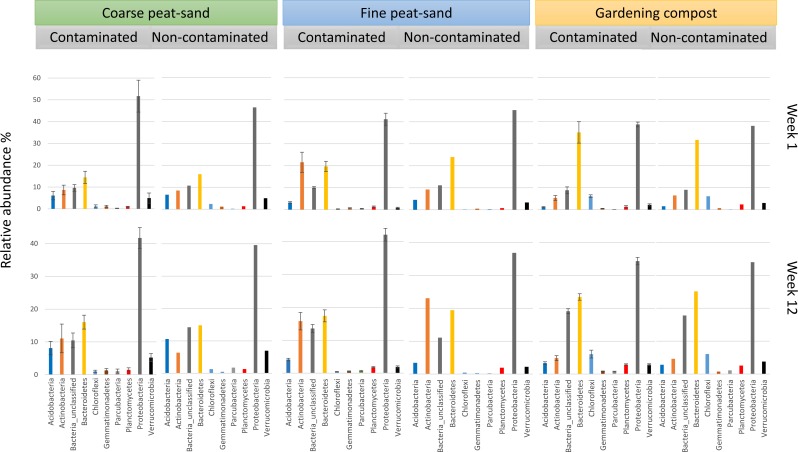
Relative abundances % at phylum level. Proteobacteria OTUs were most abundant in coarse peat-sand, fine peat-sand and gardening compost throughout the experiment.

Proteobacteria OTUs were most abundant in contaminated fine (37 –44%) and coarse peat-sand (38–62%) and gardening compost (33–40%) throughout the experiment (Fig. 2). Other abundant bacterial phyla (mean abundance of more than 1%) were Bacteroidetes (11–41%), Actinobacteria (4–26%) and Acidobacteria (1–11%) in three landscaping materials (Fig. 2). Within phyla Proteobacteria, Alphaproteobacteria were most abundant in fine peat-sand (44–58%) and gardening compost (30–37%) throughout the experiment. In coarse peat-sand, Alphaproteobacteria were more abundant at week 12 (34–43%), while Gammaproteobacteria dominated in the week 1 samples (29–53%). The most abundant bacterial class within phyla Bacteroidetes in all soil types at week 1 was Sphingobacteriia (49–63% in coarse peat-sand, 38–59% in fine peat-sand and 78–90% in gardening compost). At week 12, Sphingobacteriia were still the dominant class in coarse peat sand (74 –91%). However, Flavobacteriia was the most abundant identified class within Bacteroidetes in the fine peat-sand (10–27%) and gardening compost (12–28%).

At the higher taxonomic levels, the three most abundant classified genera in the contaminated coarse peat-sand were *Pseudomonas*, *Arthrobacter* and *Terrimonas,* and *Arthrobacter*, *Rhodanobacter* and *Nitrospira* in the fine peat-sand, throughout the experiment (Table S8). The three most abundant classified genera in the gardening compost were *Aequorivita*, *Devosia* and *Flavobacterium* (Table S8), but the most abundant bacteria were unclassified Bacteroidetes (13 ± 2%), Chloroflexi (3 ± 1%) and other bacteria (5 ± 2%). From the six most abundant classified bacteria genera *Arthrobacter* and *Devosia* were abundant in all three landscaping materials (Table S8).

### Temporal changes in bacterial community composition in landscaping materials

**Figure 3:**
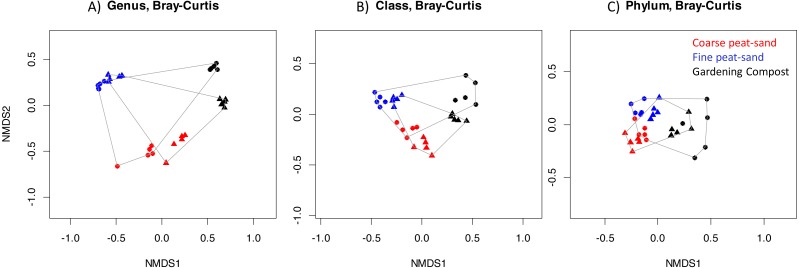
Temporal changes in the bacterial community composition. Bacterial community composition differed between contaminated landscaping materials and between weeks 1 and 12: (A) Genus level. (B) Class level. (C) Phylum level. Timepoints shown are: Circle, week 1; Triangle, week 12.

To determine temporal changes in bacterial communities, we compared bacterial community compositions between weeks 1 and 12 in contaminated landscaping materials. Bacterial community compositions differed between all three tested landscaping materials and between weeks 1 and 12 from OTU to phylum level (PERMANOVA: *p* < 0.001, Fig. 3, Table S9). In addition, community composition of Actinobacteria, Bacteroidetes and Proteobacteria (including Proteobacteria classes) differed between landscaping materials and weeks (PERMANOVA: *p* < 0.001, Table S9).

**Diversity and richness changes.** OTU diversity and richness were lower in fine peat-sand at week 1 compared to week 12 (Paired *t*-test: *p* < 0.001, Table S9). The changes in fine peat-sand involved increased diversity of Actinobacteria, Bacteroidetes, Proteobacteria, Alpha- and Gammaproteobacteria from week 1 to week 12 (Paired *t*-test: *p* < 0.01, Table S9). There were no diversity or richness changes at the OTU level in coarse peat-sand and in gardening compost, but diversity of Bacteroidetes and Deltaproteobacteria in coarse peat-sand and diversity of Gammaproteobacteria in gardening compost decreased during the experiment (Paired *t*-test: *p* < 0.05, Table S9).

**Relative abundance changes.** Relative abundance of Deltaproteobacteria was increased from week 1 to week 12 in all of the three landscaping materials (Paired *t*-test: *p* < 0.02, Table S9), whereas relative abundance of Betaproteobacteria was decreased in coarse peat-sand and in gardening compost (Paired *t*-test: *p* < 0.005, Table S9). Relative abundance of Bacteroidetes, Proteobacteria and Gammaproteobacteria decreased in gardening compost (*p* < 0.01), whereas in fine peat-sand the relative abundance of Actinobacteria was decreased (*p* < 0.02) and the relative abundance of Gammaproteobacteria increased from week 1 to week 12 (*p* < 0.01) (Paired *t*-test: Table S9).

At a higher taxonomic level, the relative abundance of *Rhodanobacter* decreased from week 1 to week 12 in all three landscaping materials (Table 3). In coarse peat-sand and in gardening compost, the relative abundance of *Aerimonas* and *Flavobacterium* decreased during the experiment (*p* < 0.05 and *p* < 0.001, respectively, Table 3). In fine peat-sand these abundances increased, but the relative abundances of these two genera were very low in fine peat-sand both in week 1 and 12 (Table 3). The relative abundance of *Arthrobacter* decreased both in fine peat-sand and in gardening compost and the relative abundance of *Pseudolabrys* decreased both in fine and coarse peat-sands (Table 3). In addition, temporal changes in gardening compost involved an increase in the relative abundances of *Gemmatimonas*, *Opitutus, Pseudolabrys* and *Terrimonas*, and decrease of *Aequorivita*, *Methylophilus* and *Pedobacter* (Table 3). In fine peat-sand, temporal changes additionally involved increase in the relative abundance of *Nitrosospira* and *Pseudomonas* and decrease in the relative abundance of *Devosia,* whereas the relative abundance of *Devosia* increased in coarse peat-sand (Table 3). One unclassified genus within the phylum Bacteroidetes and another within the class Gammaproteobacteria were increased in coarse (*p* < 0.05) and fine peat-sands (*p* < 0.01), whereas unclassified genus within family Xanthomonadaceae was decreased (*p* < 0.01) and unclassified genus within order Myxococcales was increased in coarse peat-sand (*p* < 0.01) and in gardening compost (*p* < 0.05) (Paired *t*-test: Table S9).

**Table 3:** Relative abundances differed between week 1 and 12. According to *t*-tests results, relative abundances of abundant genera (abundance over 1%) changed during the experiment.

	Coarse peat-sand				Fine peat-sand				Gardening compost			
Genus	Mean week 1	Mean week 12	*t*	*p*-value	Mean week 1	Mean week 12	*t*	*p*-value	Mean week 1	Mean week 12	*t*	*p*-value
*Acinetobacter*	40 ± 49	15 ± 24	0.8	0.464	1 ± 1	25 ± 23	−2.0	0.110	108 ± 26	87 ± 101	0.4	0.680
*Aequorivita*	3 ± 2	0.2 ± 0.4	2.5	0.065	3 ± 2	2.4 ± 2	0.2	0.884	252 ± 80	91 ± 9	4.5	0.011
*Arenimonas*	121 ± 19	66 ± 25	3.7	0.022	1 ± 1	5 ± 2	−3.3	0.031	24 ± 3	17 ± 4	6.4	0.003
*Arthrobacter*	185 ± 41	175 ± 85	0.2	0.843	667 ± 181	361 ± 74	4.2	0.014	146 ± 32	91 ± 21	4.6	0.010
*Devosia*	81 ± 13	166 ± 37	−4.0	0.017	112 ± 11	92 ± 15	4.7	0.009	173 ± 17	164 ± 17	0.6	0.605
*Flavobacterium*	137 ± 26	13 ± 8	7.8	0.001	13 ± 5	22 ± 5	−2.9	0.045	245 ± 31	59 ± 7	12.3	0.000
*Gaiella*	83 ± 21	78 ± 18	0.4	0.701	77 ± 21	66 ± 11	0.8	0.489	1 ± 1	2 ± 1	−1.4	0.242
*Gemmatimonas*	71 ± 28	84 ± 32	−0.6	0.582	43 ± 12	37 ± 6	1.5	0.198	33 ± 6	56 ± 5	−4.3	0.012
*Methylophilus*	117 ± 36	89 ± 22	1.2	0.308	1 ± 1	2 ± 2	−1.3	0.256	68 ± 9	45 ± 6	3.8	0.019
*Nitrosospira*	8 ± 3	11 ± 2	−1.7	0.163	55 ± 19	136 ± 14	−7.0	0.002	1 ± 1	1 ± 1	0.4	0.704
*Opitutus*	133 ± 72	154 ± 43	−0.6	0.595	13 ± 5	24 ± 10	−2.3	0.081	58 ± 15	94 ± 15	−3.5	0.026
*Pedobacter*	23 ± 3	10 ± 8	2.6	0.059	79 ± 8	95 ± 14	−1.7	0.163	224 ± 39	53 ± 9	9.1	0.001
*Pseudolabrys*	70 ± 16	39 ± 6	3.9	0.017	101 ± 11	75 ± 10	2.9	0.045	4 ± 3	9 ± 3	−4.4	0.012
*Pseudomonas*	748 ± 560	260 ± 235	1.5	0.199	22 ± 11	63 ± 29	−4.4	0.012	115 ± 19	40 ± 18	7.5	0.002
*Rhodanobacter*	15 ± 5	4 ± 2	5.3	0.006	191 ± 21	139 ± 32	3.1	0.038	80 ± 14	17 ± 4	11.4	0.000
*Terrimonas*	96 ± 45	224 ± 115	−2.5	0.068	1 ± 1	1 ± 0	0.0	1.000	16 ± 5	48 ± 7	−7.7	0.002

### Bacterial community composition distances between contaminated and non-contaminated materials

Bacterial community composition distances (Bray-Curtis) were analyzed between contaminated (five replicates) and non-contaminated (a non-replicated composite sample from five subsamples) samples within each landscaping material. This resulted in five distances per landscaping material within each time point. Average distances were thereafter compared between different landscaping materials, and between the two time-points (weeks 1 and 12) within each landscaping material. The reasoning was to determine potential alterations associated with contamination.

**Figure 4:**
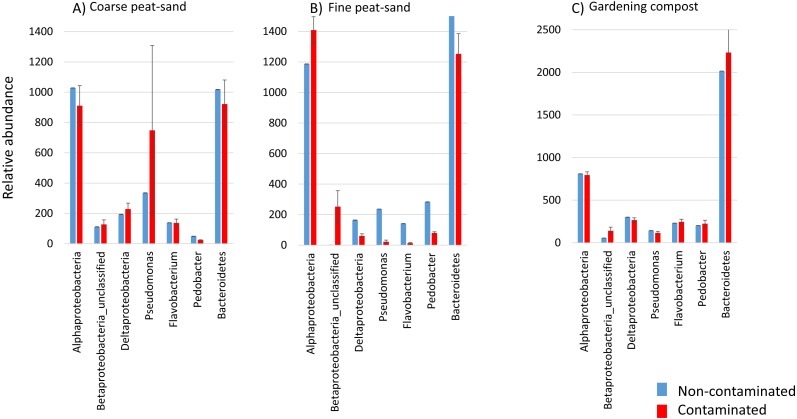
Relative abundances of bacterial phylotypes. Relative abundance of seven bacteria phylotypes at week one are shown here in non-contaminated (blue) and contaminated (red) landscaping materials: (A) Coarse peat-sand. (B) Fine peat-sand. (C) Gardening compost.

Long bacterial community composition distances were observed particularly in fine peat-sand at week one (Table S5). At this time point, the relative abundance of unclassified Betaproteobacteria genus was high and Deltaproteobacteria, *Pseudomonas*, *Flavobacterium* and *Pedobacter* abundances were low in contaminated fine peat-sand compared to non-contaminated material (Fig. 4). In fine peat-sand, bacterial community composition distance at OTU level was 0.58 ± 0.01 (Mean ± SD) at week 1, whereas at week 12 this same distance was 0.39 ± 0.04. At different taxonomic levels, average bacterial community composition distances in fine peat-sand varied between 0.14–0.77 at week 1 and 0.10–0.49 at week 12 (Table S5). In coarse peat-sand, distances varied between 0.09–0.48 and 0.11–0.52 at weeks 1 and 12, and in gardening compost between 0.06–0.42 and 0.04–0.36, respectively (Table S5).

Short bacterial community composition distances were observed at phylum level in all three landscaping materials (Mean variation at week 1 and 12 0.04–0.14, Table S5), indicating that contamination may not affect bacterial community composition at the phylum level. In contrast, the longest community composition distances at week one in coarse and fine peat-sands were observed within class Deltaproteobacteria (0.48 ± 0.10 and 0.77 ± 0.04, respectively). In addition, Betaproteobacteria community composition distances were long at week 1 both in fine peat-sand (0.66 ± 0.06) and in gardening compost (0.42 ± 0.03) compared to week 12 (0.32 ± 0.03 and 0.34 ± 0.05, respectively). At week 12, the longest community composition distance was observed within class Betaproteobacteria in coarse peat-sand (0.52 ± 0.16), whereas in fine peat-sand longest community composition distances were observed within classes Gammaproteobacteria (0.49±0.10) and Deltaproteobacteria (0.48 ± 0.07), and in gardening compost within class Alphaproteobacteria (0.36 ± 0.02) (Table S5).

## Discussion

### Connections between PAH degradation, bacterial community composition and temporal microbiota changes

We aimed to find potential associations between bacterial community composition and PAH degradation. In this study, higher abundances of Beta-, Delta- and Gammaproteobacteria were associated with a shorter half-life of pyrene. In addition, higher diversity of Bacteroidetes and Betaproteobacteria was associated with a shorter half-life of pyrene. However, there might be reasons (see ‘Connections between half-lives and properties of landscaping materials’ and ‘Connections between half-lives and molecular weight’) other than bacterial degradation ability that affected the half-life of pyrene. This is evident, since half-life of pyrene is short also in sandy gravel with undetectable bacterial sequence reads, whereas chrysene was very persistent in sandy gravel and higher relative abundance of Betaproteobacteria was associated with shorter half-life of chrysene. Betaproteobacteria abundance was higher in coarse peat-sand and gardening compost at week 1 when PAH concentrations were higher compared to week 12. In addition, in coarse peat-sand, where half-life of chrysene was shortest, Betaproteobacteria community composition had longest distance between contaminated and non-contaminated material compared to other distances at week 12. Based on previous studies, Betaproteobacteria may be an important indicator for the bioremediation process of PAHs (Lors et al., 2010) and the primary phenanthrene degraders in soil (Martin et al., 2012). In addition, in soils with high pyrene concentration, Betaproteobacteria abundance may increase, whereas Deltaproteobacteria abundance may concurrently decrease (Ren et al., 2015). In agreement with these findings, we have observed a higher relative abundance of the Betaproteobacteria and a lower relative abundance of the Deltaproteobacteria in the creosote (includes ∼85% PAH) contaminated soils as compared to non-contaminated soils in our previous study (Parajuli et al., 2017).

In this study, temporal microbiota changes concentrated on dominant phyla and classes that have previously been associated with soil pollution, and to genera which abundance was more than 1%. Synchronous temporal microbiota changes in all three landscaping materials involved increase in relative abundance of Deltaproteobacteria as PAH levels decreased during the experiment. In contrast, the relative abundance of Betaproteobacteria decreased with decreasing PAH concentrations in coarse peat-sand and gardening compost. *Rhodanobacter* (Gammaproteobacteria) was the sole genus in which relative abundance decreased during the experiment in all three landscaping materials. *Rhodanobacter* genus includes denitrifying acid-tolerant bacteria (Green et al., 2012) that has been associated with PAH degradation in some studies (Kanaly, Harayama & Watanabe, 2002; Song et al., 2016; Li et al., 2017). However, *Rhodanobacter* genus involvement in *in situ* degradation remains questionable, and it seems that the genome lacks functional genes associated with PAH degradation (Li et al., 2017). Another genus for which the relative abundance decreased considerably during the experiment was *Flavobacterium* (Bacteroidetes). This decrease was obvious in coarse peat-sand and gardening compost. In fine peat-sand, the overall bacterial diversity and richness, and the diversity of Actinobacteria, Bacteroidetes and Proteobacteria, particularly Alpha- and Gammaproteobacteria, were lower at week 1 compared to week 12, whereas the diversity of Bacteroidetes and Deltaproteobacteria in coarse peat-sand, and the diversity of Gammaproteobacteria in gardening compost, decreased during the experiment. Decreased diversities might reflect the effect of the PAH contamination; previous studies have also reported that PAH contamination decreases bacterial diversity (Mukherjee et al., 2014; Ren et al., 2015).

We additionally hypothesized that PAH concentrations typical to urban soils adjust microbial communities in commonly used landscaping materials and these changes differ between landscaping materials. In support of this hypothesis, we detected long distances between contaminated and non-contaminated fine peat-sand after one-week of exposure to PAHs. At this time point, the longest distances were observed within classes Beta- and Deltaproteobacteria (0.66 ± 0.06 and 0.77 ± 0.04, respectively) in fine peat-sand. Interestingly, the bacterial community composition distances of Beta- and Deltaproteobacteria seems to be associated with PAH contamination in all landscaping materials included in our study; this observation is in line with earlier investigations showing that these two classes are typically related to PAH contamination in soil (Ren et al., 2015; Parajuli et al., 2017). However, it must be taken into account that we used a composite non-contaminated sample that represents bacterial composition at a community-wide scale, and we used this composite sample to calculate Bray-Curtis distances between non-contaminated and contaminated landscaping materials. On the one hand, this method can reduce variability in heterogeneous samples and detect the dominant phylotypes of the bacterial community (Manter, Weir & Vivanco, 2010). On the other hand, this method can influence the estimates of phylotype richness and diversity, and locally dominant but spatially rare phylotypes may become rare in the final sample (Manter, Weir & Vivanco, 2010). In support of our study findings, the phylotypes that we observed to have long distances between non-contaminated and contaminated samples include the same phylotypes that were detected in previous studies with PAH contamination (Singleton et al., 2006; Lors et al., 2010; Martin et al., 2012; Mukherjee et al., 2014; Ren et al., 2015; Parajuli et al., 2017). Additionally, the phylotypes that we observed to have long distances between contaminated and non-contaminated materials were the same as those associated with pyrene or chrysene degradation, i.e., Beta-, Delta- and Gammaproteobacteria.

After twelve-week follow-up, PAHs concentrations were lower and bacterial communities had greater similarity between contaminated and non-contaminated fine peat-sand. It is currently uncertain whether community composition changes are permanent or temporary in real urban environments where PAH deposition is continuous. However, previous research has revealed that the abundance of Betaproteobacteria may be greater in urban parks than in forests under evergreen trees (Hui et al., 2017). This might be the consequence of the PAH deposition in urban parks, since Betaproteobacteria are associated with the PAH contamination in this and other studies (Lors et al., 2010; Martin et al., 2012; Mukherjee et al., 2014; Ren et al., 2015; Parajuli et al., 2017).

### Connections between half-lives and properties of landscaping materials

We also hypothesized that the half-life of PAHs correlates with substrate properties, such as organic matter content and pH of the landscaping material. Half-lives of phenanthrene, fluoranthene and pyrene were considerably lower in landscaping materials with low organic contents of 1–2% (1.5–4.4 weeks) (sandy gravel and coarse peat-sand) than in fine peat-sand with organic content of 13% (2.8–52 weeks) and gardening compost with organic content of 56% (2.5–36 weeks). However, the organic matter content was unrelated to the half-life of pyrene. High organic matter content can decrease the bioavailability, degradation and diffusion of PAHs by increasing the sorption of PAHs to the organic fractions (Ran et al., 2007; Yang et al., 2010; Luo et al., 2012). Thus, one reason for the longer half-lives for phenanthrene, fluoranthene and pyrene in fine peat-sand and in gardening compost might be the sorption of PAHs to the organic matter, while physico-chemical characteristics of each PAH and bacterial degradation ability may also have influences. Chemical persistence and physical properties of PAHs affect the biodegradation rate since bacteria are known to degrade only water dissolved compounds, and water solubility decreases and solid/water distribution ratio increases when the complexity of PAHs increases (Johnsen, Wick & Harms, 2005).

Both gardening compost and fine peat-sand had a carbon-to-nitrogen ratio of 25:1, which may have negatively impacted the biodegradation effectiveness, since a ratio of 10:1 has been shown to be more optimal (Teng et al., 2010). Optimal carbon-to-nitrogen ratio offers enough nutrients for microbial growth, which may enhance the biodegradation (Teng et al., 2010). In addition, there were differences in the nutrient concentrations between landscaping materials, which might have influenced the biodegradation effectiveness, since bioavailable nutrients may stimulate PAH degrading bacteria (Chaudhary et al., 2012). Bacterial community composition can also be affected by pH gradient (Mukherjee et al., 2014). However, pH was not correlated with PAH half-lives in this study.

### Connections between half-lives and molecular weight

Our goal was to estimate how much PAH degradation depends on the number of aromatic rings. Generally, a great diversity of bacteria can degrade the low molecular weight PAHs, but relatively few bacterial genera degrade PAHs with more than four aromatic rings, such as benzo[b]fluoranthene (Juhasz & Naidu, 2000; Folwell, McGenity & Whitby, 2016). Thus, high molecular weight PAHs are more resistant to biodegradation (Johnsen, Wick & Harms, 2005; Haritash & Kaushik, 2009). Our results corroborate with these findings: benzo[b]fluoranthene was resistant to biodegradation and chrysene was very persistent in sandy-gravel and its half-life was over 17-weeks longer in coarse peat-sand and over 19-weeks longer in gardening compost than the half-lives of lower molecular weight PAHs. In fine peat-sand, the half-lives of pyrene (52 weeks) and chrysene (49 weeks) were almost the same, and approximately 10-weeks longer than the half-life of fluoranthene and over 46-weeks longer than the half-life of phenanthrene. Interestingly, the half-life of pyrene was 20-weeks shorter in gardening compost than the half-life of fluoranthene. This might be the effect of PAH mixture, since the degradation of PAH mixtures may be more multifaceted than degradation of individual compounds (Johnsen, Wick & Harms, 2005). With PAH mixtures, degradation of phenanthrene, fluoranthene and pyrene can be inhibited (Hennessee & Li, 2016), but phenanthrene can also enhance the degradation of higher molecular weight PAHs (Mclellan, Warshawsky & Shann, 2002).

In sandy gravel, commonly used in urban landscaping (Morel, Chenu & Lorenz, 2015), persistence of PAHs with molecular weights ≥228 g mol^−1^ was high, resulting in the accumulation of higher molecular weight PAHs in urban soils and greater risks to human health. Long half-life of chrysene (198 weeks) confirms the persistence of chrysene in sandy gravel, but a first-order kinetic model is more appropriate for low molecular weight PAHs during a 12-weeks study (Crampon et al., 2014). Thus, extrapolating a half-life calculation based on 12 weeks might be inaccurate for chrysene in sandy gravel, but it is used in this study to reflect only on the persistence (Tansel et al., 2011). It is known that lower molecular weight PAHs are more volatile and phenanthrene, fluoranthene and pyrene exist in the gas and particle phase, whereas chrysene and benzo[b]fluoranthene exist exclusively in particle phase (Ravindra, Sokhi & Van Grieken, 2008). Therefore, in this study it is possible that phenanthrene, fluoranthene and pyrene volatilize out of porous materials with low organic matter content (low sorption to organic particles) like sandy gravel and coarse peat-sand. However, we did not quantify volatilization. Yet our study does not exclude the possible degradation of low molecular weight PAHs in sandy gravel despite the undetectable bacterial sequence reads. Preventing the volatilization of PAHs is important for human health. For example, fluoranthene is an experimental carcinogen and occurs at high concentrations in urban air (Boström et al., 2002).

### Potential associations between bacterial community changes and health-associated bacteria

The “altered environmental microbiome” hypothesis, as an explanation for how environmental pollution is associated with microbiota and health, was first introduced in our previous study (Parajuli et al., 2017). Bacterial community changes, i.e., reduced bacterial diversity and altered Bacteroidetes and Proteobacteria communities, caused by PAH pollution (current study and others—Singleton et al., 2006; Lors et al., 2010; Martin et al., 2012; Peng et al., 2013; Mukherjee et al., 2014; Sawulski, Clipson & Doyle, 2014; Ren et al., 2015; Parajuli et al., 2017) include changes in bacteria potentially connected to immune-mediated diseases (Abrahamsson et al., 2012; Hanski et al., 2012; Knip & Siljander, 2016; Zhou & Zhi, 2016; Santoru et al., 2017). PAH pollution often explains abundances of Beta- and Gammaproteobacteria (Martin et al., 2012; Peng et al., 2013; Mukherjee et al., 2014; Ren et al., 2015) and these two classes may have a potential role in the early priming of the immune system (Ruokolainen et al., 2015). Decreased abundance of genus *Flavobacterium* within phyla Bacteroidetes in the human gut have been associated with a risk of inflammatory bowel disease, which is a chronic inflammatory disease of the gastrointestinal tract, including ulcerative colitis and Crohn’s disease (Santoru et al., 2017). In addition, decreased abundance of *Bacteroides* have been associated with a risk of inflammatory bowel disease (Zhou & Zhi, 2016; Santoru et al., 2017) and IgE-associated eczema (Abrahamsson et al., 2012). In contrast, Bacteroidetes dominance and the greater abundance of the genus *Bacteroides* have been associated to type 1 diabetes risk (De Goffau et al., 2013; Knip & Siljander, 2016).

Our findings suggest that diversity of bacteria, particularly Bacteroidetes and Proteobacteria, may decrease with increased PAH concentration. Reduced overall bacterial diversity in the human gut is associated with type 1 diabetes (Knip & Siljander, 2016), eczema (Abrahamsson et al., 2012; Ismail et al., 2012) and asthma (Abrahamsson et al., 2014). In addition, low genetic diversity of Gammaproteobacteria on the skin is associated with an increased risk of atopy and decreased expression of IL-10 which is an anti-inflammatory cytokine (Hanski et al., 2012). Because PAH contamination can change bacterial communities in polluted soils, it is also possible that PAH contamination can also directly affect the human microbiota. Bacteria, especially Proteobacteria, in the human gut contribute to a variety of biological functions, i.e., immune modulation and metabolism (Bradley & Pollard, 2017), and reduced bacterial functional diversity in the gut microbiome increases the risk of type 1 diabetes (Brown et al., 2011). Connecting bacterial community composition changes in PAH-contaminated landscaping materials at the laboratory scale and changes in human gut microbiota in patients with immunological disorders is highly hypothetical and we encourage further studies. In addition, it is unclear to what extent immune-mediated diseases are the cause and to what extent they are the consequence of bacterial community composition changes. Further studies are needed to determine how bacterial functions are affected during PAH contamination and to examine the possible associations between urban PAH pollution and microbial community changes both in the living environment and in the human gut. Such studies are especially important in children, because the immune system develops in early childhood.

### Recommendations to manage urban environments in the context of human health

As urban populations grow, the quality of the urban environment will play an important role in public health (WHO, 2015). In industrialized countries, approximately 78% of the human population lives in urban areas with little green space and limited exposure to natural biodiversity (United Nations, 2014). According to the biodiversity hypothesis, this reduced contact with natural, resident microbial biodiversity negatively affects the human commensal microbiota and immune system (Hanski et al., 2012). Evidence exist that microbial communities in urban environments differ from those in rural environments, and that urbanization reduces the transfer of diverse environmental microbiota indoors (Pakarinen et al., 2008; Qian et al., 2017; Parajuli et al., 2018).

Two of the greatest challenges of urban societies are ecologically sustainable management of urban spaces, particularly soils and green areas (Morel, Chenu & Lorenz, 2015), and the control of rapidly increasing prevalence of immune-mediated diseases (Hanski et al., 2012). As a solution, we propose research targeting the development of novel gardening and landscaping materials that both speed up the degradation of PAHs and support the development of the human immune system. It must also be considered that vegetation in urban parks affects the soil microbiota (Hui et al., 2017) and effectiveness of PAH degradation (Haritash & Kaushik, 2009), and sometimes landscaping materials are mixed with other soil materials when used in urban areas. Novel gardening and landscaping materials could be designed to harbor biodiverse non-culturable microbial communities of forest floor to fill the gap in immunomodulation of urban dwellers that face only a fraction of the environmental microbiota present in natural and many rural ecosystems.

## Conclusions

Bacterial community composition, organic matter content and molecular weight of PAHs affects the half-life of PAHs in landscaping materials. We found a relationship between higher relative abundance of Betaproteobacteria and shorter half-lives of pyrene and chrysene. In addition, higher relative abundances of Gamma- and Deltaproteobacteria were correlated with shorter half-life of pyrene. High organic matter content (≥13% in this study) might decrease the degradation efficiency by increasing the sorption of PAHs to organic fractions, whereas coarse-grained landscaping materials with low organic matter content (1–2%) might increase the probability of volatilization of PAHs with a molecular weight ≤202.

Temporal microbiota changes were particularly evident within class Deltaproteobacteria, and genera *Flavobacterium* and *Rhodanobacter*. Relatively low PAH contamination seems to adjust Proteobacteria community composition, particularly Beta- and Deltaproteobacteria communities. PAH contamination may also result in a decreased bacterial diversity. Bacterial community composition alterations include changes in bacteria phyla associated with human health and immune-mediated diseases.

##  Supplemental Information

10.7717/peerj.4508/supp-1 Table S1 Characteristics of landscaping materials In this study, we used four different landscaping materials: sandy gravel, coarse peat-sand, fine peat-sand and gardening compost. Organic matter content (OM), pH, water holding capacity (WHC) and degree of coarseness (DC) were determined from five replicates, and nutrients and elements from one replicate. a. Mean ± SD, b. Manufacturer declared, c. LOQ = 50 µg l ^−1^ extract, d. LOQ carbon = 0.10%, LOQ nitrogen = 0.12%, e.LOQ = 3.5.

10.7717/peerj.4508/supp-2 Table S2 Chemical characteristics of investigated PAHs and their manufacturers Landscaping materials were contaminated with PAH mixture containing phenanthrene, fluoranthene, pyrene, chrysene and benzo[b]fluoranthene. AR, Number of aromatic rings, MW, Molecular weight g mol ^−1^ (Oleszczuk & Baran, 2003), S, Aqueous solubility (g m3 ^−1^) (Oleszczuk & Baran, 2003), Ultra, Ultra Scientific, Kingstown, RI, USA, Dr.Ehr., Dr. Ehrenstorfer GmbH, Ausburg, Germany.

10.7717/peerj.4508/supp-3 Table S3 Chemical data Concentrations and recoveries for PAHs and surrogate standard recoveries.

10.7717/peerj.4508/supp-4 Table S4 Bacterial data DNA was analyzed for bacterial (16S) communities at weeks 1 and 12 using Illumina MiSeq metabarcoding.

10.7717/peerj.4508/supp-5 Table S5 Bacterial relative abundances, diversities, richness and distances Diversity (the Shannon index), richness (the Chao index) and the relative abundance of dominant bacteria phyla, class and genera, and bacterial community composition distance (Bray-Curtis) between contaminated and non-contaminated materials.

10.7717/peerj.4508/supp-6 Table S6 Multiple comparison Multivariate analysis of variance (MANOVA) with Bonferroni correction was used to compare half-life of PAHs (log transformed) in different landscaping materials.

10.7717/peerj.4508/supp-7 Table S7 Regression statistics The linear regression models were used to estimate the relationship between half-lives and properties of the landscaping materials, and between half-lives and bacteria relative abundances and diversities.

10.7717/peerj.4508/supp-8 Table S8 The six most abundant classified bacteria genera in landscaping materials Relative abundance % of the six most abundant bacterial genera in the three of the studied contaminated landscaping materials (Mean of the week 1 and 12 ± SD).

10.7717/peerj.4508/supp-9 Table S9 Permanova and *t*-test statistics PERMANOVA was used to compare temporal differences in the bacterial community composition between weeks 1 and 12. Differences in the richness (the Chao index), diversity (the Shannon index) and relative abundances between weeks 1 and 12 were determined using the paired *t*-test.

## Additional Information and Declarations

### Data Availability

The following information was supplied regarding data availability: Sequence Read Archive Accession SRX3195289–SRX3195338 (Study SRP117895).
