# Supplementary material for: Half-lives of PAHs and temporal microbiota changes in commonly used urban landscaping materials
Source: PeerJ. 2018 Mar 19;6:e4508. doi: 10.7717/peerj.4508 (PMC5863720; doi:10.7717/peerj.4508)
Supplement: Table S1 — In this study, we used four different landscaping materials: sandy gravel, coarse peat-sand, fine peat-sand and gardening compost. Organic matter content (OM), pH, water holding capacity (WHC) and degree of coarseness (DC) were determined from five replicates, and nutrients and elements from one replicate. a. Mean ± SD, b. Manufacturer declared, c. LOQ = 50 µg l −1 extract, d. LOQ carbon = 0.10%, LOQ nitrogen = 0.12%, e.LOQ = 3.5. [file peerj-06-4508-s001.docx]

|  | **Sandy gravel** | **Coarse peat-sand** | **Fine peat-sand** | **Gardening compost** |
| --- | --- | --- | --- | --- |
| **Soil type** | Glacial sand | Technologically processed mull | Fertilized and limed mull | Composted and limed mull |
| **Soil material** | Crashed and sieved coarse mineral aggregate fraction | Coarse mineral soil and peat | Fine-grained mineral soil and peat | Garden peat, broiler manure, compressed wood fiber, crashed bark |
| **Additional ingredients** | No | No | Organic fertilizer of animal origin, lime and manufactured humic acid | Limestone dust with magnesium |
| **OM % ^a^** | 1 ± 0.1 | 2 ± 0.1 | 13 ± 0.7 | 56 ± 1.2 |
| **pH ^a^** | 6.4 ± 0.2 | 5.6 ± 0.1 | 5.4 ± 0.3 | 6.7 ± 0.1 |
| **WHC % ^a^** | 18 ± 0.8 | 22 ± 1.2 | 43 ± 3.0 | 73 ± 2.3 |
| **DC^b^, mm** | 0-36 | 31 | 16 | < 35 |
| **Nitrate^c^** | <LOQ | 11.0 | 294 | 618 |
| **Ammonium** | 0.1 | 0.4 | 0.7 | 16 |
| **Phosphate** | 0.1 | 0.2 | 17 | 25 |
| **Nitrogen^d^ %** | <LOQ | <LOQ | 0.2 | 1.3 |
| **Carbon^d^ %** | <LOQ | 0.5 | 5.3 | 32.1 |
| **Phosphorus** | 715 | 248 | 622 | 1936 |
| **Iron** | 13302 | 5445 | 11631 | 6128 |
| **Aluminum** | 8754 | 3656 | 6880 | 5854 |
| **Manganese** | 181 | 66 | 138 | 244 |
| **Copper** | 18 | 12 | 21 | 15 |
| **Lead^e^** | <LOQ | <LOQ | 4 | <LOQ |
