# Supplementary material for: Half-lives of PAHs and temporal microbiota changes in commonly used urban landscaping materials
Source: PeerJ. 2018 Mar 19;6:e4508. doi: 10.7717/peerj.4508 (PMC5863720; doi:10.7717/peerj.4508)
Supplement: Table S2 — Landscaping materials were contaminated with PAH mixture containing phenanthrene, fluoranthene, pyrene, chrysene and benzo[b]fluoranthene. AR, Number of aromatic rings, MW, Molecular weight g mol −1 (Oleszczuk & Baran, 2003), S, Aqueous solubility (g m3 −1) (Oleszczuk & Baran, 2003), Ultra, Ultra Scientific, Kingstown, RI, USA, Dr.Ehr., Dr. Ehrenstorfer GmbH, Ausburg, Germany. [file peerj-06-4508-s002.docx]

| **PAHs** | **Chemical formula** | **AR** | **MW** | **S** | **Manufacturer** |
| --- | --- | --- | --- | --- | --- |
| **Phenanthrene** | C_14_H_10_ | 3 | 178 | 1.29 | Ultra |
| **Fluoranthene** | C_16_H_10_ | 4 | 202 | 0.26 | Ultra |
| **Pyrene** | C_16_H_10_ | 4 | 202 | 0.14 | Dr. Ehr. |
| **Chrysene** | C_18_H_12_ | 4 | 228 | 0.002 | Dr. Ehr. |
| **Bentzo[b]- fluoranthene** | C_20_H_12_ | 5 | 252 | 0.0012 | Ultra |
