# Supplementary material for: Half-lives of PAHs and temporal microbiota changes in commonly used urban landscaping materials
Source: PeerJ. 2018 Mar 19;6:e4508. doi: 10.7717/peerj.4508 (PMC5863720; doi:10.7717/peerj.4508)
Supplement: Table S8 — Relative abundance % of the six most abundant bacterial genera in the three of the studied contaminated landscaping materials (Mean of the week 1 and 12 ± SD). [file peerj-06-4508-s008.docx]

| **Genera** | **Relative abundance %** | **Class** | **Phyla** |
| --- | --- | --- | --- |
| **Coarse peat-sand** | | | |
| ***Pseudomonas*** | 7.7±8 | Gammaproteobacteria | Proteobacteria |
| ***Arthrobacter*** | 2.8±1.3 | Actinobacteria | Actinobacteria |
| ***Terrimonas*** | 2.2±1.7 | Sphingobacteriia | Bacteroidetes |
| ***Opitutus*** | 1.7±0.9 | Opitutae | Verrumicrobia |
| ***Devosia*** | 1.6±0.8 | Alphaproteobacteria | Proteobacteria |
| ***Methylophilus*** | 1.4±0.5 | Betaproteobacteria | Proteobacteria |
| **Fine peat-sand** | | | |
| ***Arthrobacter*** | 8±3.3 | Actinobacteria | Actinobacteria |
| ***Rhodanobacter*** | 2±0.3 | Gammaproteobacteria | Proteobacteria |
| ***Nitrosospira*** | 1.4±0.6 | Betaproteobacteria | Proteobacteria |
| ***Pseudolabrys*** | 1.3±0.3 | Alphaproteobacteria | Proteobacteria |
| ***Devosia*** | 1±0.2 | Alphaproteobacteria | Proteobacteria |
| ***Pedobacter*** | 1.2±0.2 | Sphingobacteriia | Bacteroidetes |
| **Gardening compost** | | | |
| ***Aequorivita*** | 2.5±1.5 | Flavobacteriia | Bacteroidetes |
| ***Devosia*** | 1.9±0.2 | Alphaproteobacteria | Proteobacteria |
| ***Flavobacterium*** | 1.7±1.1 | Flavobacteriia | Bacteroidetes |
| ***Arthrobacter*** | 1.6±1.5 | Actinobacteria | Actinobacteria |
| ***Pedobacter*** | 1.5±1.4 | Bacteroidetes | Sphingobacteriia |
| ***Acinetobacter*** | 1.5±1.2 | Gammaproteobacteria | Proteobacteria |
